# Human Herpesviruses 6A and 6B in Reproductive Diseases

**DOI:** 10.3389/fimmu.2021.648945

**Published:** 2021-03-25

**Authors:** Anthony L. Komaroff, Roberta Rizzo, Jeffrey L. Ecker

**Affiliations:** ^1^Division of General Medicine, Department of Medicine, Brigham and Women’s Hospital, Harvard Medical School, Boston, MA, United States; ^2^Department of Chemical and Pharmaceutical Sciences, University of Ferrara, Ferrara, Italy; ^3^Department of Obstetrics, Gynecology and Reproductive Biology, Massachusetts General Hospital, Harvard Medical School, Boston, MA, United States

**Keywords:** congenital infection, human herpesvirus-6A, human herpesvirus-6B, inherited chromosomally integrated human herpesvirus 6, preeclampsia, primary unexplained infertility, spontaneous abortion, intrauterine growth retardation (IUGR)

## Abstract

Human herpesviruses 6A (HHV-6A) and human herpesvirus 6B (HHV-6B)—collectively, HHV-6A/B—are recently-discovered but ancient human viruses. The vast majority of people acquire one or both viruses, typically very early in life, producing an ineradicable lifelong infection. The viruses have been linked to several neurological, pulmonary and hematological diseases. In early human history, the viruses on multiple occasions infected a germ cell, and integrated their DNA into a human chromosome. As a result, about 1% of humans are born with the full viral genome present in every cell, with uncertain consequences for health. HHV-6A may play a role in 43% of cases of primary unexplained infertility. Both the inherited and acquired viruses may occasionally trigger several of the factors that are important in the pathogenesis of preeclampsia. Transplacental infection occurs in 1-2% of pregnancies, with some evidence suggesting adverse health consequences for the child. While emerging knowledge about these viruses in reproductive diseases is not sufficient to suggest any changes in current practice, we write this review to indicate the need for further research that could prove practice-changing.

## Introduction

Human herpesvirus-6A (HHV-6A) and human herpesvirus-6B (HHV-6B)—collectively, HHV-6A/B—are ancient human viruses that were discovered only about 30 years ago (1, 2). An expanding group of human illnesses have been definitively or provisionally linked to the viruses (3, 4).

Recently, studies have indicated that HHV-6A may be one cause of unexplained primary infertility and that both HHV-6A and HHV-6B may, in some cases, contribute to the pathogenesis of preeclampsia (PE). If the viruses play a role in these conditions, it may be due to their ability to infect endometrial epithelial cells, placental cells, natural killer (NK) cells and the endothelial cells of myometrial spiral arteries—and due to the immune response to infection. Both viruses also can cause transplacental infection of the newborn.

We will briefly describe the biology of these viruses. Then, we will describe what is known about their relationship to primary infertility and to PE, as well as what is known about transplacental congenital infection of newborns and its possible health consequences.

## Biology of HHV-6A and -6B

HHV-6A and HHV-6B are betaherpesviruses, members of the *Roseolovirus* genus, as described in more detail elsewhere (3, 5–7). Initial infection with HHV-6B occurs in early childhood, and somewhat later for HHV-6A. Infection of the respiratory tract, including the tonsils and olfactory-ensheathing cells of the nasal cavity, is the primary route of infection (5, 8). Horizontal transmission, particularly from adult to child, is likely (7). Primary infection from breast feeding or blood transfusions has not been reported (3).

HHV-6A/B infects a wide variety of cells and tissues including: 1) multiple immune system cells—including CD4+ T cells, CD8+ T cells and NK cells; 2) multiple cells of the nervous system—astrocytes, microglial cells, oligodendrocytes and neuronal cells; 3) and cells of other tissues—liver cells, human fibroblasts, epithelial cells and endothelial cells (4, 6). The viruses also can infect multiple cells of the reproductive tract (9), as will be discussed shortly.

Over 95% of adults are infected with HHV-6B. A smaller but substantial fraction are infected with HHV-6A. As with all herpesviruses, the infections are permanent: the viruses establish latency—a state in which they cannot be eradicated and from which they can periodically reactivate (7).

Infection with HHV-6B in early childhood can cause roseola infantum (exanthem subitem) (10), cause minimal symptoms or be asymptomatic. HHV-6B causes encephalitis/encephalopathy and delirium in people undergoing hematopoietic stem cell transplantation (11–14). The viruses are a common trigger for febrile seizures in young children, including febrile status epilepticus (15–21). As summarized in detail elsewhere, they may be one trigger of mesial temporal lobe epilepsy; multiple sclerosis; drug rash with eosinophilia and systemic symptoms (DRESS); and Hashimoto’s thyroiditis (4).

When the viruses are acquired by a person early in life, they permanently infect a small number of *somatic* cells, inserting their full genomes into the telomeric region of a host cell’s chromosomes, through molecular mechanisms recently identified (22–24).

Remarkably, and of potential importance in reproductive disease, on multiple occasions in human history, both viruses inserted their genomes into a human *germ* cell. The earliest known occurrence of this appears to have been between 85,000-342,000 years ago, in Africa (25). Consequently, about 1% of the human race is born with the entire viral genome inside every cell—a condition called inherited chromosomally-integrated HHV-6A/B (iciHHV-6A/B) (22, 26, 27). This *inherited* viral genome can be transcriptionally active, producing viral proteins and even full virions. Diagnosis of iciHHV-6A/B can be made by viral load studies in whole blood that exceed 5.5 log10 copies/ml (26). Investigators have begun to explore the health consequences of iciHHV-6A/B (28).

**Figure 1:**
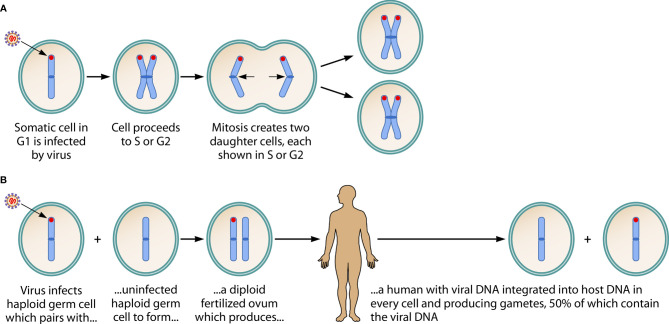
Consequences of HHV-6A/B DNA integration into a chromosome of a somatic cell, and into the host chromosome of a germ cell. **(A)** Shows acquired virus integrating its DNA (in red) into the telomere of a chromosome of a *somatic* cell. It has been hypothesized that this is a mechanism by which HHV-6A/B achieves latency. Since the viral genome is not integrated into the DNA of sperm or ova, no vertical transmission of the viral genome occurs. (Note: This figure shows only one of the 23 chromosomes, in early metaphase, and assumes viral infection and integration occurs in the G1 phase, then is replicated in the S phase and passed to both of the daughter cells during mitosis. When the integration event occurs during S or G2 phases—not shown in the figure—the daughter cells create a mosaic, since one contains the viral genome and the other does not.) **(B)** Describes an ancient event: on several occasions in human history, the viral genome integrated into the DNA of a haploid *germ* cell chromosome. This led to a fertilized ovum containing the viral genome and, hence, to a human with the viral genome integrated into a chromosome in *every* cell: inherited chromosomally-integrated HHV-6 (iciHHV-6). In Mendelian fashion, the integrated viral DNA is present in 50% of gametes (whether sperm or ova). About 1% of humans are born with iciHHV-6. The *inherited* presence of the viral genome in *every* cell, including *germ* cells, contrasts with the integration of *acquired* virus into only a small fraction of target *somatic* cells. *Adapted from, and reprinted with permission of the publisher, from: Komaroff AL, Pellett PE, Jacobson S. Human herpesvirus 6A and 6B in brain diseases: Association vs. causation. Clinical Microbiology Reviews. 2021;* 34:e00143-20. https://doi.org/10.1128/CMR.00143-20.

The infection of *somatic* cells by *acquired* virus, and the ancient infection of a *germ* cell leading to an *inherited* viral genome, are summarized in **Figure 1**.

## HHV-6A/B in Reproductive Organs

### Prevalence of HHV-6A/B

HHV-6A and -6B can be found in the vaginal canal, cervix, and the uterus (29, 30). HHV-6A/B DNA has been detected in genital tract secretions from about 4% of non-pregnant women (30–32) and about 2-18% of pregnant women (29, 32–34), with higher viral loads in pregnant women (32, 34). It also is detected in about 10% of women attending a sexually transmitted disease clinic (31). In the great majority of cases, the infection is acquired rather than inherited.

### Cellular Targets of HHV-6A/B

#### Infection of Endometrial, Cervical and Placental Cells

Most pertinent to their possible role in reproductive diseases, HHV-6A can infect endometrial cells (35) and syncytiotrophoblasts (35, 36). The virus reaches the reproductive organs *via* the circulation (primarily, infected lymphocytes). It is unclear if the virus can be transmitted sexually. The viruses also can infect cervical carcinoma cell lines, and have been identified along with human papillomavirus as a coinfecting agent in some cervical carcinoma tissue (37).

#### Infection of Endometrial NK Cells

HHV-6A/B infection of immune system cells can lead to the production of various chemokines (38) and chemokine receptors (6) as well as pro-inflammatory cytokines including IL-1β, TNF-α, IF-α, IF-γ and IL-6 (39–44). It also can decrease the expression of anti-inflammatory cytokines such as IL-10 (45).

The NK cell, in particular, plays a potentially important role in both primary infertility and PE. NK cells are the dominant lymphocytes in the endometrium. HHV-6A (and -6B) can infect both an NK cell line (46) as well as primary NK cells (47).

In a *non-pregnant* woman, when endometrial epithelial cells are infected by HHV-6A, the NK cytotoxic attack is severe, leading to the production of proinflammatory cytokines that inhibit implantation (47). Thus, viral infection of endometrial NK cells is a plausible contributor to the pathogenesis of primary infertility.

In a *pregnant* woman, in contrast, NK cells are less prone to attack “foreign” antigens. When NK cell receptors recognize human leucocyte antigen-E (HLA-E) and HLA-G antigens on cytotrophoblasts, the NK cells do not attack cells containing paternal antigens, facilitating implantation (48). Theoretically, HHV-6A/B infection of NK cells could disrupt the down-regulation of the NK cell attack on cytotrophoblasts, contributing to defective implantation and to the pathogenesis of both primary unexplained infertility and PE.

#### Infection of Endothelial Cells

Finally, HHV-6A/B also can infect endothelial cells (49–51), presumably including the endothelial cells of the myometrial spiral arteries, impairing implantation. It is important that this possibility be investigated.

## Primary Unexplained Infertility

### Evidence Linking HHV-6A to Infertility

Investigators examined endometrial biopsy tissues from 30 women with unexplained primary infertility (no evidence of endometriosis, endometritis, recurrent miscarriage, ovulatory dysfunction or anatomical uterine pathologies) and 36 fertile women with at least one previous successful pregnancy. The two groups were similar with regard to age, length of menstrual cycle, smoking habits, and levels of FSH, LH, TSH, FT4 and progesterone (9).

HHV-6A (but not HHV-6B) DNA was found in the endometrial epithelial cells of 43% of women with primary unexplained infertility vs. none of the fertile controls, a highly significant difference (P<0.00001). *In vitro* studies demonstrated that endometrial NK cells in the infertile, infected women attacked endometrial epithelial cells that were infected with the virus (9).

### Pathobiology of Infertility

One likely cause of primary unexplained infertility is defective endometrial receptivity (52, 53). Inflammatory changes in the endometrium and/or in the placenta, triggered by infection, theoretically could inhibit implantation. Indeed, viral agents long have been postulated as possible environmental factors in infertility (54, 55).

### Endometrial HHV-6A Infection

HHV-6A infection of endometrial epithelial cells, endometrial NK cells (46, 47) and trophoblast cells (35, 36) promotes changes that can impair implantation. Indeed, women with primary unexplained infertility and endometrial HHV-6A infection have higher levels of pro-inflammatory cytokines in uterine washings (56).

HHV-6A infection of the endometrium reduces levels of two decidualization markers, soluble human leucocyte antigen-G (sHLA-G) and mucin1 (48, 57), possibly augmenting the maternal immune response against paternally-derived fetal antigens (56). Infection of endometrial epithelial cells also generates a pattern of microRNA expression that has been linked to implantation failure (35).

HHV-6A infection of the endometrium in women with primary unexplained infertility is seen more often in women with a particular polymorphism for an ATP-gated ion channel, P2X7R. An antagonist of P2X7R has been shown to reduce the infectability of target cells by HHV-6A, and to reduce its replication, *in vitro* (56).

HHV-6A infection of endometrial epithelial cells inhibited the ability of a human choriocarcinoma trophoblast cell line to attach to endometrial cells (35). Although HHV-6A also can infect syncytiotrophoblast cells *in vitro* (35, 36), it has not been demonstrated that such infection occurs *in vivo*, and influences implantation.

### Summary

HHV-6A infection of both endometrial epithelial cells, endometrial NK cells and, possibly, trophoblasts may be one trigger of primary unexplained infertility, through multiple different mechanisms that may impair implantation. Further research is required to determine if therapeutic interventions directed at blocking the pathobiology produced by endometrial HHV-6A infection—for example, antiviral therapy—improve prognosis in women with primary unexplained infertility, particularly in those with documented endometrial infection. If antiviral therapy improved prognosis, it would strongly suggest an etiologic role for HHV-6A.

## HHV-6A/B and Preeclampsia (PE)

### Evidence Linking HHV-6A/B to Some Cases of Preeclampsia

Investigators from the University of Cambridge looked for non-human messenger RNA (any RNA virus or replicating DNA virus) in placental samples from 99 cases of PE [according to ACOG 2013 criteria (58)], 48 cases of fetal growth retardation and 132 healthy pregnancy controls (59). Since the study involved placentas obtained at term, it could not assess whether active viral infection *earlier* in the course of a pregnancy might contribute to the etiology of PE. Importantly, the investigators had no prior hypothesis about any particular virus that might be linked to PE: they used nucleic acid sequencing to detect any known human virus present.

The analysis revealed only two viruses to be correlated with PE: HHV-6A/B. Quantitation of the viral DNA, and study of the DNA from both parents, confirmed that in 70% of the positive cases the viral genes had been *inherited* from either the mother or father (iciHHV-6A/B), and that in 30% of cases the virus had been *acquired* from the mother. Analysis of the inherited DNA sequence and the mRNA sequences found in the placenta confirmed that the inherited viral DNA was transcribing the viral mRNA found in the placenta (59).

The investigators then sought to replicate their findings in two larger datasets. In the first, the presence of iciHHV-6A/B was determined in cord blood DNA from 368 pregnancies complicated by PE and 3,674 pregnancies without PE. When these subjects were combined with the original cases and controls, iciHHV-6A/B was found in 2.1% of cases vs. 0.8% of controls (OR 2.8, P=0.008) (59).

The investigators then studied cord blood from 740 new cases of PE and compared the incidence of iciHHV-6A/B in these cases to the incidence in a meta-analysis of several large-scale population studies involving 61,549 subjects. The incidence in cases and controls was 1.6% vs. 0.7% (OR 2.5, P=0.001) (59).

If iciHHV-6A/B increases the probability of PE, one might expect it also to increase the probability of spontaneous abortion—through some of the mechanisms discussed below. Indeed, a recent report of 23 women with iciHHV-6A/B compared to 285 matched controls without iciHHV-6A/B finds a significantly increased odds ratio of spontaneous abortion (OR 6.41, 95%CI 1.10-37.4) (60). Theoretically, iciHHV-6A/B also might increase the probability of intrauterine growth retardation, a possibility that requires investigation.

Hence, iciHHV-6A/B—and, possibly, acquired infection with HHV-6A/B—appear over-represented among cases of PE, and may therefore predispose to PE. At the same time, HHV-6A/B are found only in a small fraction of cases—at least as determined by tissue (placentas, cord blood) obtained at *term*. Since studies of the virus in endometrial tissue *earlier* in pregnancy might conclude the viruses are linked to a larger fraction of cases, this possibility should be investigated.

### Pathobiology of PE

If acquired or inherited HHV-6A/B increases the risk of PE, they most likely do so through one or more of the many well-established risk factors for PE. Indeed, it is plausible that HHV-6A/B could affect several of those risk factors.

#### Abnormal Placentation

Whereas implantation is prevented or ended prematurely in infertility, it is allowed but distorted in miscarriage and PE (53). While abnormal placentation may not be a *necessary* first step in the pathogenesis of PE, it may well be a contributing factor in women with other risk factors for PE—particularly hypertension, diabetes or obesity (61).

Defective placentation may lead to placental ischemia due to a failure of proliferating cytotrophoblasts to penetrate the decidua, invade the myometrium, and remodel the myometrial spiral arteries into the wide, low-pressure vascular channels necessary for a healthy pregnancy. While HHV-6A/B can infect syncytiotrophoblasts (35, 36), it is unclear if they can infect cytotrophoblasts [as can another betaherpesvirus, cytomegalovirus (62)], and thereby inhibit the capacity of cytotrophoblasts to form a robust villous architecture and to remodel myometrial spiral arteries.

Finally, as discussed earlier, HHV-6A/B infection of NK cells could also lead to defective implantation and consequent placental ischemia, contributing to the pathogenesis of PE.

#### Endothelial Dysfunction, Atherosclerosis, Thrombosis

There is systemic and placental dysfunction of the vascular endothelium in PE, manifested by impaired vasodilation (63–65), and increased reactivity to angiotensin II (66). Endothelial dysfunction, in turn, likely contributes to several key features of PE: hypertension, proteinuria, edema and coagulopathy. HHV-6A/B can infect endothelial cells, provoking endothelial dysfunction, atherosclerosis (49–51, 67) and the intravascular inflammation (increased immune cellular infiltrates and cytokine levels) seen in PE (68). The relevance of endothelial infection by HHV-6A/B to PE requires further study.

#### Defective Angiogenesis

An excess of antiangiogenic forces relative to angiogenic forces is seen in PE. This is reflected by increased plasma levels of soluble endoglin (sEng) plus an increased ratio of soluble tms-like tyrosine kinase 1 (sFlt-1)/placental growth factor (PIGF), which are strongly predictive of PE (68–70). As of now, it is not clear whether and how HHV-6A/B might contribute to defective angiogenesis, beyond its apparent ability to promote endothelial dysfunction.

#### Genetic Factors in PE

Twin studies find that PE has a heritability range from 22% to 47% (68), and that the inherited vulnerability may derive either from the maternal or paternal side.

For decades, the literature on PE has spoken of “dangerous fathers”: 1) men associated with one previous pre-eclamptic pregnancy who are more likely to be associated with a future pre-eclamptic pregnancy with a new partner (61); and 2) men born from a PE pregnancy, whose future spouse(s) are at higher risk of developing PE (61). Some of the risk from “dangerous fathers” comes from fathers with thrombophilias or polymorphisms in the *IGF2* gene; however, these factors are not found in all “dangerous fathers”, indicating that other factors also may be involved (61).

Thus, the University of Cambridge study raises the question of whether one of those as-yet unidentified factors producing “dangerous fathers” may be iciHHV-6A/B: the greatly increased risk of PE seen in that study was the same whether the carrier of iciHHV-6A/B was the mother or the father (59).

## HHV-6A/B Congenital Infection

HHV-6A/B can cause “congenital HHV-6A/B infection”, as defined by the presence of HHV-6A/B DNA in cord blood or placental tissues (71). About 1-2% of normal neonates have congenital HHV-6A/B infections; in 10% of congenital HHV-6A/B infections there are elevated viral loads in the infant’s blood; one third are due to HHV-6A (30, 72–74).

Congenital HHV-6A/B infection can occur in three ways: 1) iciHHV-6A/B in a parent is passed to the infant, with the infants inheriting the viral genome in every cell—which is the cause of 86% of cases of congenital infection; 2) in a mother with iciHHV-6A/B, the inherited viral genome produces viruses that are passed transplacentally to the infant—even though the infant does not have iciHHV-6A/B (i.e., the haploid oocyte that produced the infant did not contain the integrated viral genome)—which is the cause of about 10% of cases of congenital infection; 3) the mother has acquired (not inherited) the virus and then passed it transplacentally to the infant—which is the cause of about 4% of cases of congenital infection, as occurs with cytomegalovirus (74, 75).

While it now is well established that congenital infection with HHV-6A/B occurs, it is uncommon. More important, it is unclear whether congenital HHV-6A/B infection affects the health of the baby (or mother) (74). One study did find that children with transplacental infection subsequently have significantly lower scores on the Bayley Scale of Infant Development MDI instrument (76), but additional studies of developmental and other health outcomes are needed.

## Conclusion

Human herpesviruses-6A and -6B (HHV-6A/B) are capable of infecting a remarkably wide range of cells and tissues, and are being linked to an increasing number of diseases (4). We summarize evidence that they may be linked to reproductive diseases, as are multiple other viruses (e.g., rubella virus, cytomegalovirus (54, 62).

One study has found endometrial infection by HHV-6A in 43% of cases of primary unexplained infertility vs. 0% of fertile, well-matched control subjects, a highly significant difference (9). The effects of the virus on the endometrial epithelium, on endometrial NK cells and possibly trophoblasts make the observed association plausible, and suggest the viruses could plausibly play a role in spontaneous abortion and intrauterine growth retardation.

The inherited form of infection (iciHHV-6A/B), and possibly the acquired form, are associated with PE, although only in a small fraction of cases. Effects of the viruses on placentation and endothelial function that could contribute to PE require further study, as well as whether the inherited form of the virus explains some cases of “dangerous fathers” more likely to be associated with cases of PE.

Finally, we have summarized how congenital infection can occur in 1-2% of neonates: the possible consequences for the health of the baby or mother warrant further study.

In short, we seek to alert readers to the possibility that these viruses can sometimes contribute to the pathogenesis of several reproductive diseases. Our emerging knowledge of the viruses does not justify any changes in current diagnostic testing and treatment practices for primary unexplained infertility, preeclampsia or congenital infection. However, the available evidence is provocative enough to justify further research.

## Author Contributions

RR reviewed all of the information (text and references) about the virology of HHV-6A/B and the information about endometrial infection and primary unexplained infertility. JE reviewed all the information about preeclampsia (text and references). AK drafted the manuscript, reviewed all the references, and integrated the editorial contributions of RR and JE. All authors contributed to the article and approved the submitted version.

## Funding

The HHV-6 Foundation supported the costs of publication.

## Conflict of Interest

The authors declare that the research was conducted in the absence of any commercial or financial relationships that could be construed as a potential conflict of interest.
